# Editorial Department

**Published:** 1882-01

**Authors:** 


### The Bistoury.

ELMIRA, N. Y., JANUARY, 1882.

The BISTOURY is published Quarterly, upon the first of April, July, October and January, at Fifty Cents a year in advance.

### Editorial Department.

PAY YOUR SUBSCRIPTION.—Blank enclosed, in which to send your subscription for another year. Two and three cent postage stamps received for fractions of a dollar. Do not delay your remittance but send at once. A dollar bill is a convenient remittance, and will pay for two years. Consult also our list of journals with which we club, giving you the BISTOURY without cost.

---

THE SUNDAY TIDINGS.—Since our last issue, a new Sunday newspaper has made its appearance in our city. It is a neat, well edited journal, and deserves well of the public by reason of its unsensational tone as well as for the encouragement of the most excellent young men who manage it. We wish the *Sunday Tidings* all the success it deserves—and that is much.

---

RATHER HARD.—A gentleman inquired at one of our bookstores, the other day, for "the hardest kind of a pencil." The attentive and urbane attendant handed him one bearing the letters H. H. H. "What do those three Hs mean?" inquired the purchaser. To this the seller responded, "O, that means a Hundred times Harder than H——l!" "A plenty hard enough, sir," responded the buyer, as he paid over the required twelve cents.

---

GOING TO FLORIDA.—Our good old friend and co-worker with the microscope—Dr. S. O. Gleason—has packed his rifle and microscope and departs immediately for the swamps and lagoons of Florida. The doctor will point his microscope toward the diatoms, desmids, rhizopods, and entomostrachiæ to be found in the swamps, and his rifle at the alligators, deer and wild-turkeys. We wish him abundant success in both endeavors. The microscopic society will miss his presence and jolly, interesting talks, but will be the richer in specimens upon his return.

HURRAH!—We wonder how many people know the real meaning of this exclamation. It is derived from *Hur-aj*, and means, literally, "to Paradise." Make a note of this fact, when you will be the better enabled to enjoy the misuse of the word in hearing the crowd hurrahing at a political meeting, for some chap whom they are unconsciously wishing "in paradise," when they really mean to have him elsewhere.

---

REVOLUTIONARY.—Mr. Vest, of the United States Senate, has been making a funny speech on "Woman's Rights," and a motion to refer a resolution on that subject to the Committee on "Revolutionary Claims." He gave two reasons for that reference. One, to awaken that committee, because they have now nothing to do; the other, because it is a revolutionary subject in reference to society, female duties, &c. His motion did not prevail, but nevertheless the strong-minded ladies to whom he referred in so ungallant a manner will, if we are not much mistaken, order Mr. Vest to the front in the first battle, or perhaps, "pull down that vest."

---

TRE POLITICAL PIC-NIC.—One of the most interesting and instructive contributions the BISTOURY has had for many a day, is the one prepared by the Rev. Thos. K. Beecher, Hon. H. H. Rockwell, Chas. A. Collin, Esq., and Dr. Krackowizer. It is this sort of political writing that does good—is instructive without being aggressive, and points out defects and virtues in parties without making one feel ashamed that he belongs to any of them.

The gentlemen who have so kindly responded to the several questions we propounded them, are regarded as among the very best political writers in their several parties. We commend their papers to our readers as models for political essayists.

---

A CANADIAN RESIDENCE.—Just what constitutes a domicil in Canada and a right thereby to a copyright protection in the British Dominions, is being determined—in rather a lively manner—by Mark Twain. Our Canadian friends should conduct themselves with great circumspection during Twain's "residence" among them, since it is not unlikely that he may be slyly taking notes for a companion volume to "Innocents Abroad"—and perhaps to be written in French, at that. Likely the bare possibility of such a scheme has induced the Canadians to banquet and otherwise entertain their newly-acquired citizen, as they have been doing. This circumstance exhibits most excellent discernment and diplomacy on their part.

---

THE HOLIDAYS.—When this number of the BISTOURY has reached our subscribers, the oasis in the desert of winter will have been passed. Christmas, with its pleasant family gatherings, its presents, stockings, trees and all its delightful belongings that go far to make the young happy and the old better; the holidays and the New Year all will be things of the past—1881 will give place to 1882. Out of the experience of the old year will come, to those who think, resolutions and plans for the new. Retrospection will recall the pleasures and pains of the past. Memory will bring before us the lost dead ones, and hope will buoy us up with fair prospects for the new comers of the old year.

The BISTOURY joins heartily in sympathy with all, but especially with those who start with resolutions to let "the dead past bury its dead."

The "living present" demands our attention and we must hurry, in this age, to keep up.

Let us store up that of the harvest of the dead year that will be of future use; clear away the rubbish of the past, and let our motto be "*Dum vivimus vivamus.*"

Dark hours will come and pass away, and bright ones will be as fleeting. Upon the way we fulfill our parts; in each may rest an eternal future.

---

A SICK HEADACHE.—We know of no ailment of the body that has so many provoking features about it as the one referred to. No man can be sicker—and live, or *care* to, for that matter—than the habitual sufferer from sick headache. Then, aside from the distressing sickness—the throbbing, dreadful pain in the head—the sensation as though your brain was being seared with a red hot iron, while little devils were thrusting barbed stillettos into every convolution of that aching organ—is the confounded annoyance you are subjected to when every acquaintance deems it his duty to apprise you of its *cause.* The last club supper—that you did or did not eat, is at once suggested as an undoubted cause. The glass of lager, drank last week, or the too hearty dinner partaken of some time during the past month, are dwelt upon and charged with your sickness. Inferentially, you are voted an ass, by your good friends, informed that you make yourself sick from lack of judgement in eating; and this too, after you have suffered the torturcs of the damned, and would cheerfully give your earthly posessions to rid yourself of it. What vexes one is, that he should be considered such a blamed idiot as to do the least thing that would likely bring on a sick headache. We *could* say more on this subject, but we feel our "dander rising" and won't.

---

CHARLES A. SPENCER.—This eminent optician died September 28th, at Geneva, N. Y., aged 70. Mr. Spencer's fame is world-wide, having accomplished more in improving microscopic objectives than any or all of the scientists of his time. He was a poor boy and self-educated. Commenced the study of optics in early boyhood, and made his own tools with which he produced his first lenses. He gradually rose to skill and fame through his own untiring zeal, laboring under the greatest disadvantages for lack of sufficient means to develop his theories and discoveries. He was the first to make the wide-angled objectives with which scientific men work to-day and by means of which so much benefit has been imparted to mankind, In late years, he has been very poor, never having possessed the means necessary to carry out the projects that, had they been developed, might have still further benefited the scientific world.

It is sad to reflect that Charles A. Spencer should have been permitted to die in poverty, his genius unrewarded, his great ability unrecognized by men of ample means, who could have benefited the world in aiding him.

---

STUFFING AN OWL.—In an unguarded moment, we volunteered our services to a good friend, to mount an owl. Anon, the owl came. He was a "wild, untamed beast," from the forests of Pennsylvania. The farmer, who deposited the box containing his owlship, upon the landing of our front door, we noticed, behaved in an extraordinary manner—he tumbled it out the hind end of his wagon with a pole, then lashed his team vigorously and disappeared around the corner.

The first comer to inspect the box was our neighbor's poodle. He left the locality as though fired from a gun, leaving a bloody streak and a continuous yell, in his wake. Fritz looked in upon him, about this time, and contributed a portion of his pantaloons. The man-of-all-work was directed to carry the box to the laboratory.

He proceeded so to do, but soon delegated the job to another, while he mended his thumb with a piece of court-plaster.

We will not dwell upon the casualties that overtook and almost overwhelmed us in trying to get the best of that owl. In disposition, fighting qualities and perfume, he is a combination of wood-chuck, wild-cat and skunk. His skin is so applied to his body as to warrant the belief that he was never intended as a specimen for the taxidermist's art. His flesh would defy the incisive capabilities of the hyena, and no feline creature could ever swallow the flesh and declare, upon honor, that he felt better for it. That owl, however, is mounted, and as an evidence of the taxidermist's skill, no member of our household dare approach him even now, as he sits perched upon our study door, looking down with his great yellow eyes as though anxious to fix his enormous talons in a fellow's scalp.

---

FAIRS AND FESTIVALS.—We doubt whether any enterprise can be named in which so much skill, labor, money, brains and downright hard labor is performed, for so little pecuniary returns, as in fairs and festivals conducted by ladies, for charitable purposes. These good hearted, generous women will toil over the dressing of dolls, construction of aprons, knitting hoods, mittens and the like, to be sold at the fair, until they are too tired and worn out to give any attention to their own household affairs. And for what reward ? We have known one little energetic woman to expend two dollars in cash for materials with which to dress a doll, work three days over its construction and then sell the contrivance for two dollars and a half. She realized just fifty cents for her three days' work and expended two dollars for the fun of earning that half-dollar.

We are acquainted with another equally enthusiastic woman in charitable work who expended seventy cents for material for a pair of silk mittens, worked almost unceasingly for an entire week and received two dollars for her production. Now, the question is, would it not be cheaper to pay that two dollars directly into the treasury and not do the work? The time could be more profitably spent in playing euchre, for instance, and would be a deal less laborious.

As to the festival part, all the ladies join in sending roast turkeys, mince pies, fancy cakes, and all manner of costly dishes, to the general rendezvous, the aggregate cost of which, we doubt not, would quite equal the sum received, at fifty cents a head, for the disposition of it. Yet, the festival part has its good features in that it brings the people together and permits of a social jollification that more than compensates for the time, perplexity and money expended in the projection. We, therefore, approve of the festival, but consider the toil and expenditure of money in preparing articles for a fair not only a useless work but also an unremunerating and absolutely senseless one.

Good lady managers of "The Home for the Aged" make no more "fairs." It doesn't pay. Contribute the money you expend for materials, éscape the labor, and be wise.

---

HATS FOR THE OPERA.—Recently, we attended the opera of "Patience." The title, it will be seen, did not accord with our feelings; for, we sat behind a young woman who was attired in an enormous white, plush hat. We say attired, because the hat was all we could see of her or her dress. We inspected that hat most thoroughly—we had nothing else to do, since to see the stage was simply impossible. It was exactly nineteen inches in diameter. [We had a rule in our pocket, happily, and can speak from absolute measurement.] It was surmounted with a plume—a white one, and would pass, we suppose, for having been plucked from an ostrich. We noted, however, that it was mended, enlarged and extended, by means of dexterously applied bits of plumes that were wrapped and wired thereto. So, we take especial pleasure in pronouncing that portion of the superstructure an unmitigated and wanton swindle. The tip of this reconstructed plume was so applied to the white plush disk, which it was intended to ornament, as to give it a sort of graceful vibratory, or compound jerky-oscillating movement that was extremely interesting. The wearer discovered this fact about the same time that we did, and found also that the effect was heightened by short, nervous, rotary movements of her head from side to side, as though she was expecting or looking for a spider upon her off shoulder. A sort of double action was imparted to it too, we found, when her side face was brought to view, particularly when this movement was executed with alacrity, for that brought the tip in contact with our nose, thereby adding increased vigor to the effect and causing each particular featherette of the artistic structure to tremble in an ecstacy of impermanence.

Wondering how the contrivance was fastened to the wearer, (for, we noticed that notwithstanding her untiring zeal in keeping the blamed thing in motion, it seemed as secure as though glued to a post,) we further inspected it, and found an enormous steel needle or pin, with a large glass head, that seemingly passed directly through the maiden's head and emerged upon the opposite side! This alarmed us somewhat, and stimulated us to further investigation. We wanted awfully to pull out that pin, and was upon the point of doing so, when the lady next us divined our intention and arrested our hand, at the same time whispering in our ear—"it goes through her switch and holds her hat on—if you pull it out, 'twill tumble off." We were sorry for this piece of information, for we wanted more than ever to remove that pin, while we wondered at our stupidity for supposing that the pin went through her brain, when any fellow might know that a girl that will wear such a contrivance upon her head, at an opera, hasn't got any brains to speak of.

---

RESPONSIBILITY OF THE INSANE.—Speculation is rife and opinions pronounced upon the criminal responsibility of the assassin of President Garfield. To come to a correct understanding as to this man's mental condition, or to what extent he is responsible for his crime, is by no means an easy problem. If we divest ourselves of our prejudices and ignore, if possible, the desire of the people for his conviction and punishment, we cannot fail to realize that the man, if not absolutely insane, is certainly not of sound mind.

That there *are* forms of insanity that would excuse an assassin, or at least hold him irresponsible for his act, we all know. When a hitherto fond mother suddenly becomes possessed of an impression that she must strangle her babe in order to obey a supposed Divine command, no one for a moment thinks her otherwise than insane—irresponsible. But, when a strong man, in the posession of good health, and faculties for business above the average of his fellows, deliberately plans and executes a murder, with the combined desire for revenge and notoriety, the line of demarkation between mental health and mental disease is by no means so clearly drawn. Mental responsibility is measured somewhat by the intelligence and mental cultivation of the individual. A man of a very low order of intellect and uncultivated brain is not held to that strict accountability that we are in the habit of requiring from his more fortunate fellows, in this regard. Eminent Judges have held that while some men are not considered capable of conducting their business—by reason of unsound mind—yet should they not be excused from the consequences of crimininal acts of great atrocity.

The Attorney-General of England declared recently that "a man may be deranged in his mind, his intellect may be insufficient for enabling him to conduct the common affairs of life, such as disposing of his property, or judging of the claims which his respective relations have upon him; and if he be so, the administration of the country will take his affairs into their management, and appoint to him trustees; but at the same time, such a man is not discharged from his responsibility for "*criminal acts.*"

Judge Bramwell, in 1860, in sentencing a murderer, made to him this declaration: "That you are of *unsound mind* I believe, but that is no reason why you should not be punished. I feel bound to sentence you to the same punishment as if you were sane." The Judge's thought or conviction, doubtless, was that an "unbalanced" or "unsound mind" is not necessarily devoid of the power of discrimination between right and wrong, or of realizing that sure and swift punishment will follow an atrocious and brutal crime.

In the "New Hampshire case," so frequently referred to—The State *vs.* Wier—Chief Justice Bell instructed the jury as follows: "The evidence must satisfy the jury that the party at the time of committing the act in question was insane, and that the disease is of such severity that the person is *incapable of distinguishing between right and wrong in that particular case,* and of controlling the sudden impulses of his own disordered mind; or, as the same rule has been laid down by an eminent Judge, a person in order to be punishable by law, must have sufficient memory, intelligence, reason and will to enable him to distinguish between right and wrong in regard to the particular act about to be done, to know and understand that it will be wrong, and that he will deserve punishment by committing it; to which I add, sufficient mental power to control the sudden impulses of his own disordered mind. * * * * I have been accustomed to regard as a distinguishing test of insanity, the inability to control the actions of a man's mind."

Another case, "The People *vs.* Wright," tried in Nebraska, in September, 1864, Judge Gantt's instruction to the jury contained the following: "In order to excuse the act of the accused upon such defence, the proof must satisfy the jury that the deprivation of the understanding is *total, fixed and permanent*, or that he was laboring under adventitious insanity and that during such frenzy there was *total deprivation* of understanding. It is a sound principle in the laws of social life, as well as in the enforcement of the criminal laws, of all civilized and enlightened governments that to excuse a man from punishment upon the ground of insanity, it must be proved distinctly and clearly that the accused was incapable of distinguishing right from wrong, at the time he did the act, and did not know it was an offence against God and man. If there be only a partial degree of reason, sufficient to have restrained those passions which produced the crime, if there be thought and design, a faculty to discover the difference between moral, good, and evil, at the time the offence was committed, then the accused was responsible for his act."

Now, the question to be decided is, is the assassin Guiteau excuseable under any of the heads enumerated by the wise jurists quoted? All seem to agree that the prisoner, in order to avail himself of the plea of insanity, must be so insane as to be unable to distinguish between right and wrong—unable to foresee the consequences of his crime or to discern that severe punishment will follow. Is this Guiteau's situation? Was or is he so deficient of intellect as not to be able to determine that he was about to "commit a crime against God and man?" Was he wholly "unable to control the actions of his own mind? Is the deprivation of his understanding 'total, fixed and permanent?'" If not, then, according to the testimony of these wise men of the law, he is guilty of murder, and should consequently suffer the full penalty of the law provided for such cases.

Scanning the testimony given in this case from day to day, and reading the pointed queries and sentences that are reported to have been uttered by the criminal, and keeping in view his intellectual development and capacity for business and roguery, we cannot excuse him upon the ground of imbecility or lack of brain development. That he is not strictly sane, there can be no doubt; but that his insanity is so pronounced as to excuse him for the reasons promulgated by the Judges cited, seems extremely doubtful. To acquit a man from all responsibility for committing such a crime as did Guiteau, is establishing an extremely hazardous precedent. The country is full of just such crazy people, who, without a just fear of the law, would be willing to satisfy a morbid desire for notoriety which just now seems so much to gratify the assassin named.

There must be a line drawn somewhere, to distinguish the responsible from the irresponsible insane. It would seem that the opinions of the eminent men given, make the distinction quite clear enough to be followed with safety.

